# Supplementary material for: Bioaccessibility and Antioxidant Activity of Polyphenols from Pigmented Barley and Wheat
Source: Foods. 2022 Nov 18;11(22):3697. doi: 10.3390/foods11223697 (PMC9689394; doi:10.3390/foods11223697)
Supplement: Supplementary file 1 [file foods-11-03697-s001.zip › foods-1986109-supplementary.pdf]

Supplementary Materials

Bioaccessibility and antioxidant activity of polyphenols from pigmented barley and wheat

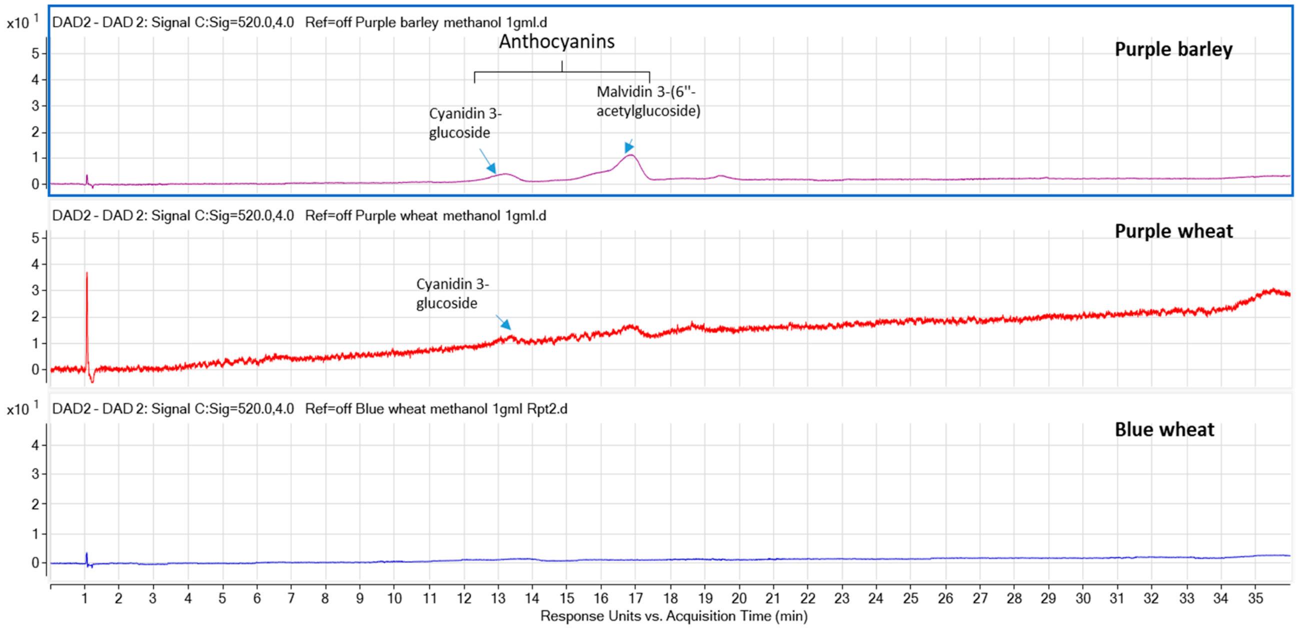

Figure S1. Chromatograms at 520 nm highlighting anthocyanin compounds in the methanol extracts of pigmented cereals. Purple line: purple barley anthocyanin profile; Red line: purple wheat anthocyanin profile; Blue line: blue wheat anthocyanin profile.

A. <u>Purple barley</u>

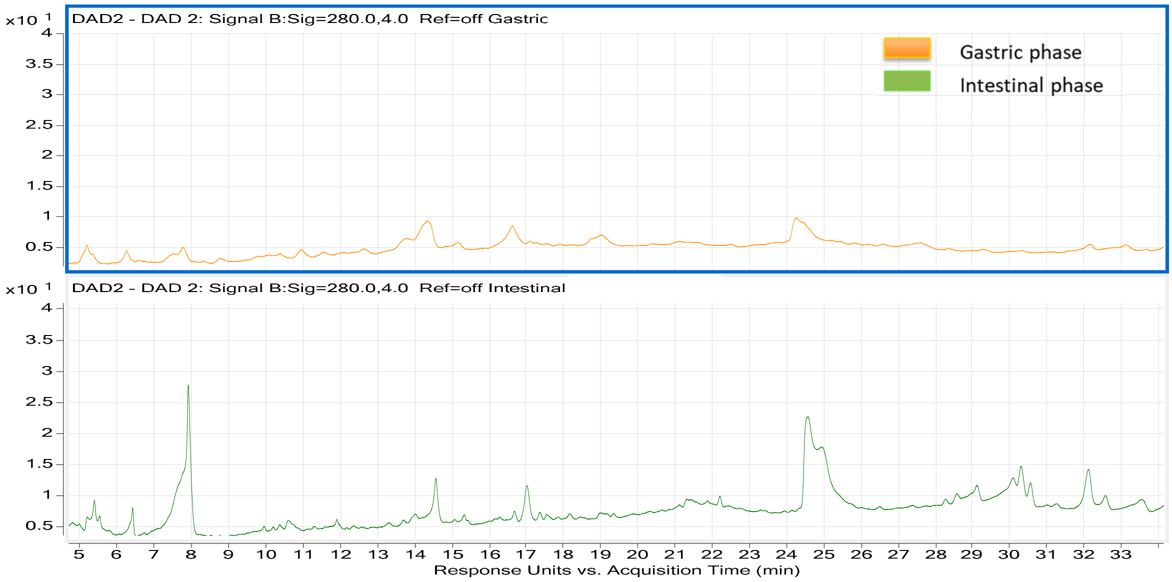

B. Purple Wheat

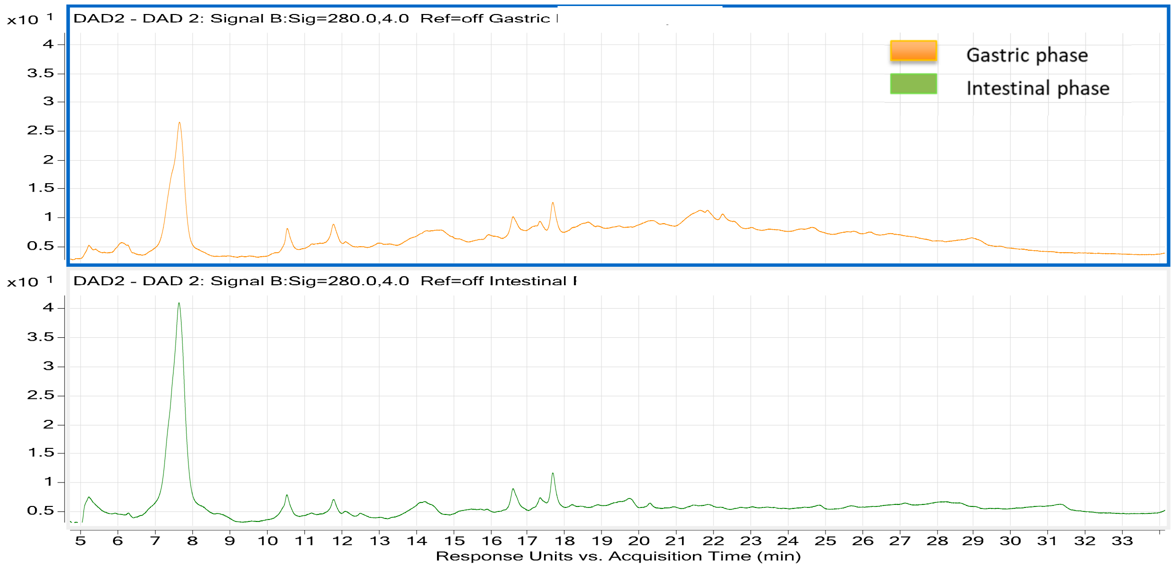

C. Blue Wheat

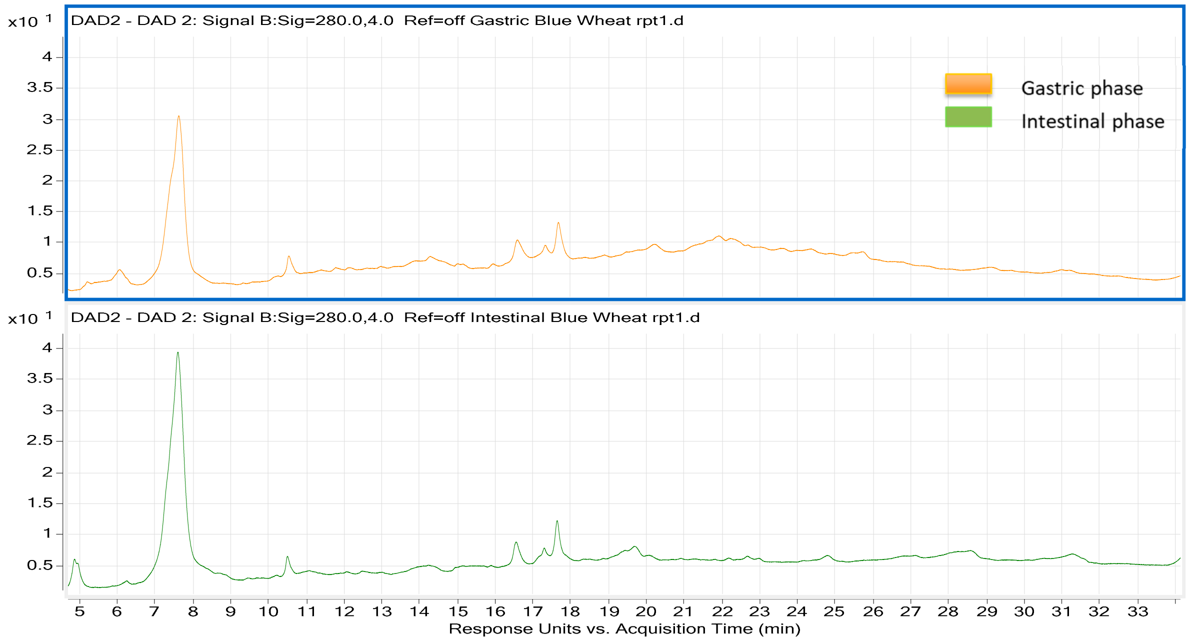

Figure S2. Annotated chromatograms of pigmented wheat flour from **gastric** and **intestinal** phases of digestion

Table S1. Table of unidentified compounds from methanol extraction

Peak	RT (mins)	Λmax (nm)	*m/z*	Phenolic quantification (mg GAE/100 g dw)		
				Purple Barley	Purple Wheat	Blue Wheat
P2	6.6	280	671.2026	0.97 ± 0[a]	0.54 ± 0[c]	0.67 ± 0.04[b]
P3	7.1	280	655.2132	–	0.06 ± 0[a]	0.06 ± 0[a]
P5 *	8.4	280	203.0839	0.33 ± 0.06[c]	5.81 ± 0.04[a]	5.39 ± 0.07[b]
P6	8.9	280	451.1238	0.04 ± 0	–	–
P8	10.5	300	323.1354	0.1 ± 0.04	–	–
P9	11.1	280	472.1578	–	0.15 ± 0.01	–
P10	11.7	255, 285	504.1444	–	0.89 ± 0.01	–
P14	14.5	280, 320	307.1393	–	0.07 ± 0.01	–
P15	14.8	320	787.3615	1.62 ± 0.27	–	–
P16	15.5	310	413.1087	–	–	0.13 ± 0
P17	15.7	320	817.3733	0.22 ± 0.02	–	–
P18	16.5	350	366.1199	–	–	0.1 ± 0.02
P21*	17.5	320	537.3072	–	–	0.13 ± 0.05
P24	20.0	320	452.2184	–	–	0.17 ± 0.01

Data are the means ± SD (n = 3). Different alphabets in each row indicates a significant difference in phenolic content. Gallic acid equivalent: GAE; Mass to charge ration: *m/z*; – not detected; RT: retention time

* P5 tentatively identified as the amino phenolic compound tryptophan based on study by (Podio et al., 2019)

* Ferulic acid was identified by mass spectra (193.0578) at 17.5 mins but not quantifiable as its UHPLC peak was masked by the other compound eluting at the same time.
